# Ultra-small-angle X-ray photon correlation spectroscopy using the Eiger detector

**DOI:** 10.1107/S1600577518013899

**Published:** 2018-10-24

**Authors:** T. Zinn, A. Homs, L. Sharpnack, G Tinti, E Fröjdh, P.-A. Douissard, M. Kocsis, J. Möller, Y. Chushkin, T. Narayanan

**Affiliations:** a ESRF – The European Synchrotron, 38043 Grenoble, France; b Paul Scherrer Institut, 5232 Villigen, Switzerland; c European X-ray Free-Electron Laser Facility, 22869 Schenefeld, Germany

**Keywords:** USAXS, XPCS, colloid dynamics, coherent X-ray scattering

## Abstract

Successful implementation of the single-photon-counting Eiger 500k pixel array detector for sub-millisecond X-ray photon correlation spectroscopy (XPCS) measurements in the ultra-small-angle scattering region is reported. The performance is demonstrated by measuring the dynamics of dilute silica colloids in aqueous solvents when the detector is operated at different counter depths, 4, 8 and 12 bit. In the fastest mode involving 4 bit parallel readout, a stable frame rate of 22 kHz is obtained that enabled measurement of intensity–intensity autocorrelation functions with good statistics down to the 50 µs range for a sample with sufficient scattering power. The high frame rate and spatial resolution together with large number of pixels of the detector facilitate the investigation of sub-millisecond dynamics over a broad length scale by multispeckle XPCS. This is illustrated by an example involving phoretic motion of colloids during the phase separation of the solvent.

## Abstract 2

The performance of the high-resolution fast-photon-counting Eiger 500k detector for sub-millisecond X-ray photon correlation spectroscopy measurement is demonstrated.

## 1. Introduction   

Scattering experiments are widely used to investigate the structure and dynamics over a broad range of size and time scales in soft matter and biological materials (Narayanan *et al.*, 2017 ▸). In particular, with the advent of high-brilliance X-ray sources over the last decades, X-ray scattering methods have become increasingly powerful (Als-Nielsen & McMorrow, 2011 ▸). The bottleneck is often the detector, which lacks the required spatial and time resolution, sensitivity and intensity dynamic range. The situation has improved remarkably with the development of hybrid pixel array detectors which enable much higher count rates and spatial resolution than gas-filled proportional counters, and higher frame rates with significantly lower noise compared with CCD-based integrating detectors (Brönnimann & Trüb, 2016 ▸). Nevertheless, limitations due to count rate, and spatial and time resolutions have not been completely resolved for many demanding scattering experiments.

A particular case is X-ray photon correlation spectroscopy (XPCS), which is a well established technique for investigating the slow dynamics in condensed matter (Grübel *et al.*, 2008 ▸; Sinha *et al.*, 2014 ▸). This method is based on the analysis of the fluctuations in the intensity of speckles emanating from the disordered microstructure within the sample upon illumination by a coherent X-ray beam (Sutton, 2008 ▸; Grübel *et al.*, 2008 ▸). Therefore, an ideal XPCS experiment should be able to resolve the individual speckles and their temporal fluctuations with single-photon sensitivity. Moreover, multispeckle analysis is essential for obtaining good statistics with low radiation dose (Vodnala *et al.*, 2018 ▸) as well as for direction-dependent studies (Leheny, 2012 ▸). These requirements set a very high demand on the performance of the detector.

In the past, significant efforts have been made to implement millisecond-range XPCS measurements using Pilatus (Westermeier *et al.*, 2009 ▸; Hoshino *et al.*, 2012 ▸) and Medipix (Caronna *et al.*, 2008 ▸; Schavkan *et al.*, 2013 ▸) pixel array detectors. These developments illustrated the feasibility of probing diffusive and glassy dynamics in particulate systems. Nevertheless, applications of multispeckle XPCS to probe sub-millisecond dynamics have been limited due to not only the available coherent photon flux from a synchrotron source but also the frame rate and resolution of two-dimensional detectors. In this respect, the Eiger single-photon-counting pixel detector developed at the Paul Scherrer Institut (PSI) is a major step forward (Dinapoli *et al.*, 2011 ▸; Johnson *et al.*, 2012 ▸). More recently, sub-millisecond multispeckle XPCS has been demonstrated using a small (128 × 256 pixels) UFXC32k hybrid pixel detector (Zhang *et al.*, 2016 ▸).

In this article, we present the performance of the Eiger 500k pixel detector for ultra-small-angle XPCS (USA-XPCS). The ultra-small-angle region provides an easier access to probe fast Brownian and advective dynamics in low viscous media (Möller *et al.*, 2016 ▸). The combination of the unique scattering vector (**q**) range (10^−3^ nm^−1^ ≤ *q* ≤ 10^−1^ nm^−1^) accessible, and the high frame rate and resolution of the Eiger detector opens a whole set of new applications. For instance, the phoretic dynamics of active colloids (Dattani *et al.*, 2017 ▸), velocity fluctuations in hydrodynamic flows (Möller & Narayanan, 2017 ▸), *etc*. can now be probed by USA-XPCS. Together with the high coherent flux from the new low-emittance multi-bend achromat lattice-based extremely brilliant synchrotron sources, it is expected that microsecond dynamics become more easily accessible by XPCS, eventually closing the gap with the neutron spin–echo technique.

The first section provides a brief outline of the detector and the readout electronics. This is followed by a description of the image acquisition scheme *via* the Library for Image Acquisition (LIMA) and integration at the beamline. Basic principles of XPCS and experimental results are presented in subsequent sections.

## 2. Detector   

### 2.1. Hardware description   

The Eiger 500k is a hybrid single-photon-counting pixel detector developed by the Swiss Light Source (SLS) Detector Group at the PSI for synchrotron applications (Dinapoli *et al.*, 2011 ▸). The detector is composed of a single sensor with eight readout chips which are organized in two halves that can be read out independently (Dinapoli *et al.*, 2013 ▸). The pixelated sensor is made of 320 µm-thick silicon and operates in the fully depletion condition. Each chip has 256 × 256 pixels of size 75 µm × 75 µm. The complete detector module consists of a sensor with 530 kpixels, corresponding to a sensitive area of about 8 cm × 4 cm, a complementary metal oxide semiconductor (CMOS) readout chip and associated electronics. Each pixel is indium bump-bonded to the individual elements of the readout chip. The depth of individual pixel counter can be software configured to 4, 8 or 12 bit, and 32 bit with on-board internal summation. The on-board memory buffers up to 2 × 4 GB of data during an acquisition sequence (*i.e.* 30000 images in 4 bit mode). The data from each half of the detector module are transferred to the control computer *via* an independent 10 Gbit s^−1^ Ethernet fiber-optic link, in addition to the 1 Gbit s^−1^ control link (Tinti *et al.*, 2015 ▸).

### 2.2. Beamline control integration   

The backend control computer is an I/O oriented Linux server whose BIOS and OS settings are tuned to high performance and low latency network transfer. The fiber-optic Ethernet links from the detector are directly terminated on this computer. The low-level detector control is performed using the *slsDetectorPackage* framework developed at the SLS. The beamline integration of the detector is realized *via* the LIMA framework for two-dimensional detector control (Petitdemange *et al.*, 2018 ▸), built on top of the *slsDetectorPackage*. The CPU and memory affinities of different tasks in the data acquisition process including the Ethernet adapter interrupt requests and packet despatching as well as image reconstruction and processing are carefully optimized by the LIMA. This allows setting different detector configurations as well as performing the data acquisition at the maximum detector hardware capabilities. The top-level configuration and acquisition commands are executed *via* the beamline control software *SPEC* (Certified Scientific Software).

### 2.3. Data acquisition   

In an experiment, the data acquisition is synchronized with the beamline control by means of a train of TTL gate signals programmed by a time frame generator (based on a compact PCI module C216). In each acquisition sequence, the number of frames, minimum latency period and exposure time were chosen according to the desired duration of the experiment and counter bit selected. In order to avoid image artifacts, it was necessary to set a minimum latency period of 20 µs which is much longer than the minimum dead-time between frames of 4 µs set by the local buffers in the readout architecture (Dinapoli *et al.*, 2011 ▸). Acquisitions of 10000 frames can be obtained at 22 kHz in 4 bit mode and frame rates reduce to 11 kHz, 6 kHz and 2 kHz in 8, 12 and 32 bit modes, respectively (Tinti *et al.*, 2015 ▸). The images are saved in ESRF data format (EDF) or hierarchical data format (HDF5) for further processing.

## 3. Experimental   

In order to demonstrate the capabilities of the Eiger 500k detector for XPCS measurements, we studied the well known Brownian dynamics of spherical colloidal particles (Berne & Pecora, 2000 ▸). In addition, we illustrate the significant improvement that has been achieved for probing faster advective dynamics using similar colloids in a phase-separating binary solvent mixture (Dattani *et al.*, 2017 ▸).

### 3.1. Materials and beamline setup   

Colloidal suspensions consisted of spherical silica particles of uniform size [diameter ∼470 nm and polydispersity index (PDI) ≃ 1.06] suspended in water at room temperature. The advective dynamics was followed using similar colloids in a phase-separating binary solvent mixture composed of 3-methylpyridine, water and heavy water.

**Figure 1:**
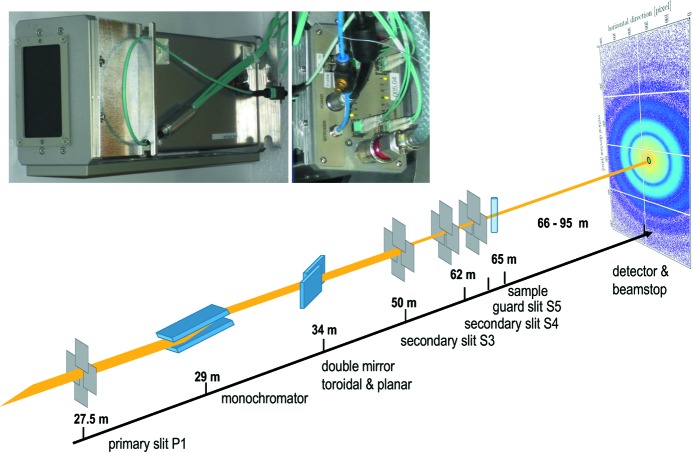
Schematic view of the beamline layout used for USA-XPCS measurements. The upper panel shows a photograph of the detector module and back-end connections.

The XPCS measurements were performed at beamline ID02 (ESRF) in the pinhole ultra-small-angle-scattering configuration using an X-ray energy of 12.46 keV (wavelength 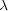 = 0.995 Å). A schematic layout of the instrument is shown in Fig. 1 ▸. The setup is optimized with a nearly coherent X-ray beam defined by two slits (S3 and S4) of size 30 µm (V) × 25 µm  (H) separated by 12 m, while P1 was closed to 0.15 mm × 0.15 mm. The strong collimation of the beam reduced the photon flux to 4 × 10^10^ photons s^−1^, which is more than three orders of magnitude lower compared with the standard small-angle X-ray scattering (SAXS) setup. Further details about the instrument can be found elsewhere (Möller *et al.*, 2016 ▸). For most of the measurements, the sample-to-detector distance was fixed to 30.7 m, which covered a *q*-range of ∼2 × 10^−3^ nm^−1^ to 10^−1^ nm^−1^, where *q* is the magnitude of the scattering vector (**q**) given by (4π/λ)sin(θ/2) with θ the scattering angle.

### 3.2. XPCS   

In a multispeckle XPCS experiment, a sequence of two-dimensional speckle patterns is recorded with exposure and lag times much shorter than the typical relaxation times probed within the sample. From the temporal fluctuations of the speckle patterns, the relevant quantity derived is the intensity–intensity autocorrelation function (Berne & Pecora, 2000 ▸),
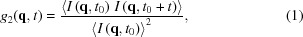
with *I*(**q**, *t*) being the scattered intensity measured at scattering vector **q** and time *t* and 〈…〉 denoting the time average. Here, *g*_2_(**q**, *t*) is related to the corresponding electric field–field autocorrelation function, *g*_1_(**q**, *t*), *via* the Siegert relation (Berne & Pecora, 2000 ▸),
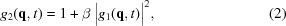
where β is the speckle contrast, which depends not only on the coherence properties of the incoming X-ray beam but also on the angular resolution of the scattering setup. In the ideal case of a perfect coherent beam and speckle size larger than the detector pixel size, β = 1. However, due to the limited coherence of the synchrotron beam and detector resolution, this factor is usually much smaller than 1 (Grübel *et al.*, 2008 ▸). The underlying dynamics of the system is manifested in *g*_1_(**q**, *t*) which in the case of pure Brownian motion follows an exponential decay,
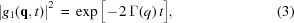
where Γ(*q*) is the *q*-dependent relaxation rate. In dilute systems, Γ(*q*) = *D*_0_ *q* ^2^, where *D*_0_ is the Stokes–Einstein diffusion constant given by *D*_0_ = *k*_B_ *T*/(6πη*R*_H_) with *k*_B_ the Boltzmann constant, *T* the absolute temperature, η the dynamic viscosity of the solvent and *R*_H_ the hydrodynamic radius of particles (Berne & Pecora, 2000 ▸).

**Figure 2:**
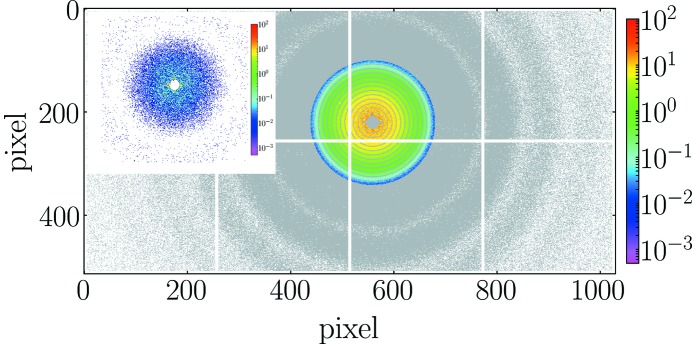
Typical time-averaged two-dimensional scattering patterns (10000 frames added) for a dilute suspension of silica particles in water. The colored area depicts the region of interest selected for the calculation of intensity–intensity autocorrelation function and chosen *q*-values are indicated as rings. The inset depicts a typical speckle pattern in the low-*q* region in a single frame with an integration time of 0.5 ms.

The XPCS data reduction was performed using the Python programme package *PyXPCS* developed at the ESRF. Each *g*_2_(**q**, *t*) function was calculated pixel-by-pixel for different **q** and then azimuthally averaged over all speckles corresponding to a given *q*-value in order to obtain the ensemble averaged *g*_2_(*q*, *t*). Fig. 2 ▸ displays the two-dimensional time-averaged scattering pattern and *q*-range used for the calculation of *g*_2_(*q*, *t*).

## 4. Results and discussion   

For the fastest XPCS measurements, the detector was operated in the 4 bit parallel readout mode at a frame rate of 22 kHz. The effective exposure time per frame was 26 µs with 20 µs latency time between each frame.

### 4.1. Brownian dynamics of colloids   

**Figure 3:**
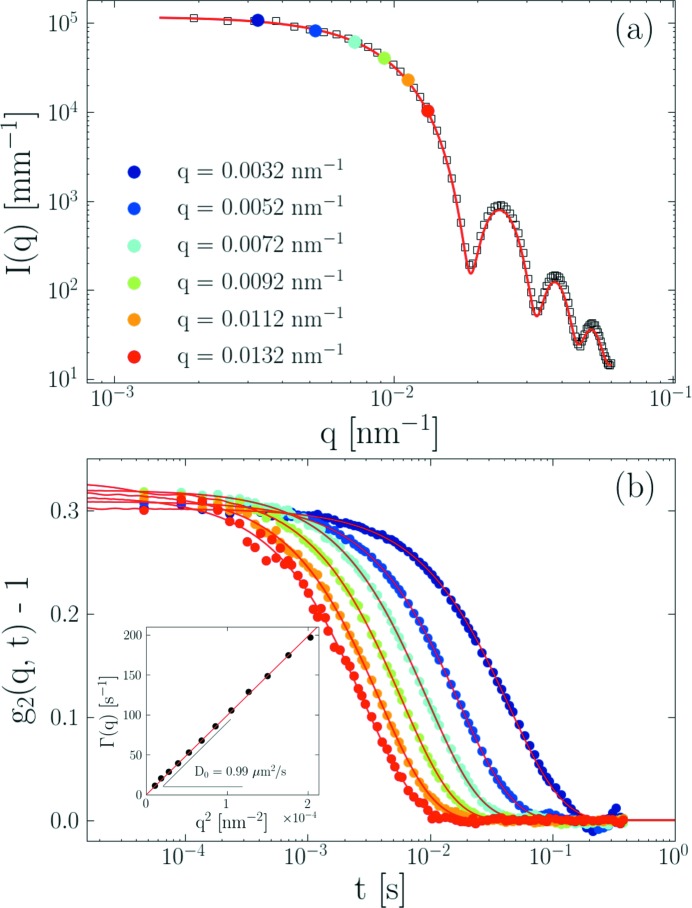
(*a*) Azimuthally averaged static scattering intensity, *I*(*q*), obtained at a sample-to-detector distance of 30.7 m for silica colloids dispersed in water together with *q*-values chosen for the determination of the intensity autocorrelation function, *g*_2_(**q**, *t*), by the *PyXPCS* programme. The continuous curve corresponds to the polydisperse sphere model with *R*_S_ = 237 nm and PDI ≃ 1.06. (*b*) Azimuthally averaged *g*_2_(*q*, *t*) for the selected *q*-values measured at 25°C. The color code is the same as that in panel (*a*). The fitted curves correspond to exponential decay given by equation (3). The inset depicts the *q*-dependence of the decay rate, Γ(*q*) = *D*_0_ *q* ^2^, with a diffusion coefficient *D*_0_ = 0.99 µm^2^ s^−1^.

This experiment was performed using dilute silica particles suspended in water. Fig. 3(*a*) ▸ depicts the azimuthally averaged static scattering profile of the particle suspension together with the selected *q*-values for which the *g*_2_(**q**, *t*) functions were calculated. In the dilute suspension, interactions between particles are negligible and the scattering features essentially represent the form factor of the spherical particles. The mean radius of particles, *R*_S_, can be directly determined from the first minimum of the form factor, *q*_min_. For spherical particles with a narrow size distribution, *q*_min_ *R*_S_ ≃ 4.483 (Narayanan, 2008 ▸). In Fig. 3(*a*) ▸, 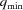 = 0.00189 nm^−1^ corresponds to *R*_S_ ≃ 238 nm, which is in perfect agreement with the value *R*_S_ = 237 nm derived from the polydisperse sphere model fit (Narayanan, 2008 ▸) shown in Fig. 3(*a*) ▸. This demonstrates the very good spatial resolution of the detector.

The *g*_2_(*q*, *t*) functions for selected *q* values are presented in Fig. 3(*b*) ▸. As shown by the solid lines, all the data can be adequately fitted using the single-exponential decay according to equation (3). The inset depicts that the decay rate, Γ(*q*), derived from the fits follow a *q* ^2^ behavior which is characteristic of a purely diffusive dynamics. The slope determined from the plot directly gives the Stokes–Einstein diffusion constant, 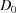 = 0.99 µm^2^ s^−1^, which corresponds to a hydrodynamic radius *R*_H_ of 247 nm using the viscosity of water 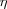 = 0.89 mPa s at 25°C. Within the uncertainty of η, the deduced value of *R*_H_ is consistent with *R*_S_ of the particles (237 nm) obtained from the polydisperse sphere model.

### 4.2. Speckle contrast   

**Figure 4:**
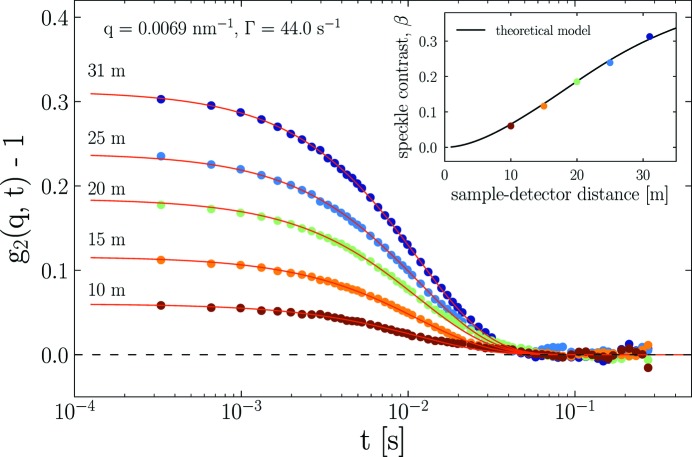
Measured *g*_2_(*q*, *t*) functions at different sample-to-detector distances for *q* = 0.0069 nm^−1^. Solid red lines represent fits to equation (2). The inset shows the variation of the speckle contrast, β, as a function of sample-to-detector distance. The theoretical model curve is calculated following Sandy *et al.* (1999 ▸) for the parameters given in the text.

The speckle contrast, β, depends not only on the degree of coherence of the incident beam but also on the effective resolution of the instrument for recording individual speckles. To illustrate this effect, XPCS measurements were performed using the silica colloidal suspension in water at different sample-to-detector distances. For this purpose, 1000 frames were acquired with an exposure time per frame of 0.23 ms and a 0.1 ms dead-time between the frames, corresponding to a total acquisition time of 0.33 s. As demonstrated in Fig. 4 ▸, β systematically reduced with a decrease of the sample-to-detector distance for a given *q* (*e.g.* 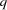 = 0.0069 nm^−1^). The different *g*_2_(*q*, *t*) functions were simultaneously fitted by equation (2) by fixing the decay rate, Γ, to the value derived at 31 m. The only free parameter was β which decreases with sample-to-detector distance due to the fact that the speckles become smaller and progressively more speckles are averaged in one pixel. This eventually leads to a very low contrast (<0.01) at the shortest measured distance of 5 m. At a sample-to-detector distance of 31 m, β is above 30% (see the inset of Fig. 4 ▸), which is more than three times larger than that obtained with the Pilatus 300k detector with a pixel size of 172 µm. It can still be increased to about 40% (*i.e*. β ≃ 0.4) by further reducing slit sizes S3 and S4 but at the expense of photon flux. The inset of Fig. 4 ▸ displays a nearly linear decrease of β with sample-to-detector distance. The theoretical model curve is calculated using the full expression for β given by Sandy *et al.* (1999 ▸) for the geometrical parameters of the scattering setup given in §3.1, the effective source size observed through the primary slit (FWHM 6 µm × 128 µm) and the magnification factor of the optics (∼2). The agreement is good for a horizontal coherence length of 11 µm.

### 4.3. Photon count statistics   

In general the speckle contrast, β, is directly obtained from equation (2). For a given *q*, β is also related to the variance of intensities within the region of the detector pertaining to that *q*. In the case of a perfect coherent source and ideal detector, β = 1, and the corresponding scattered field is a Gaussian variable. Then the probability distribution of scattered intensities, *P*(*I*), is simply given by the Rayleigh law, 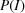 = 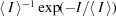 (Goodman, 1985 ▸). In the case of a partially coherent source, this distribution is not strictly followed, and the resulting *P*(*I*) is better described by a Poisson–Gamma distribution (Goodman, 1985 ▸),
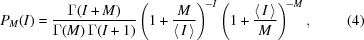
where *I* is the number of photon counts and *M* is the number of modes which defines the speckle contrast *via* *M* = 1/β.

**Figure 5:**
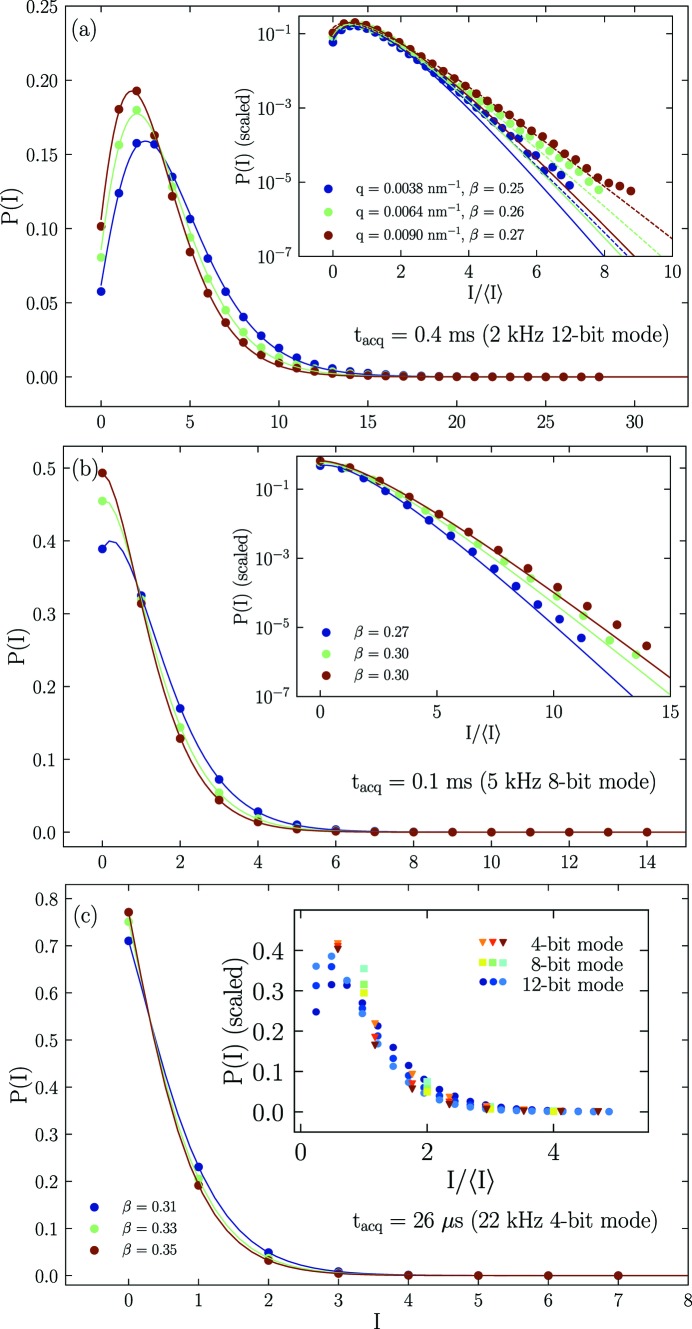
Intensity distribution functions, *P*(*I*), calculated from speckle patterns measured at 30.7 m for selected *q* values. Panels (*a*), (*b*) and (*c*) correspond to *P*(*I*) for exposure times (*t*_acq_) of 0.4 ms, 0.1 ms and 0.026 ms, respectively. Solid lines represent fit curves to equation (4). Insets display the scaled *P*(*I*) as a function of *I*/〈*I*〉. The dashed lines in the inset in (*a*) show a better agreement with equation (4) by a higher weighting of the tail region and the resulting β values are larger (∼0.33–0.36). The inset in (*c*) depicts the scaled form of whole set of data from different acquisitions.

Fig. 5 ▸ displays the typical *P*(*I*) functions calculated from speckle patterns from the silica colloidal suspension for different exposure times (in 12, 8 and 4 bit mode acquisitions). The bulk part of the distribution is described by equation (4) as indicated by the continuous lines. The variations in β derived from *P*(*I*) (given in the legends) is primarily due to the uncertainty in determining *M*. The insets of Figs. 5(*a*) and 5(*b*) ▸ present the scaled *P*(*I*) (by the area of the curve) as a function of *I*/〈*I*〉. The initial part of the curve deviates from equation (4) at low counts which may be related to the counting electronics of the detector elements. At higher photon counts, observed probabilities are larger than expected, which do not seem to be influencing the XPCS results, as the intercepts and decay rates obtained are similar. The apparent deviation at high counts can be reduced by a higher weighting of the fit in this region as indicated by the dashed lines in the inset of Fig. 5(*a*) ▸. Furthermore, at higher counts an exponential tail similar to the Gaussian statistics persists down to many orders of magnitude in *P*(*I*). This suggests that a very high degree of coherence is achieved in the setup as also indicated by the higher β value of ≥0.3. The inset in Fig. 5(*c*) ▸ displays the scaled data for all three acquisitions and the data roughly superimpose except at low counts due to different counting times or 〈*I*〉. Although, the apparent value of β obtained from *P*(*I*) is smaller at higher counts, the *g*_2_(*q*, *t*) functions show similar intercepts at different 〈*I*〉 values.

### 4.4. Fast advective dynamics   

**Figure 6:**
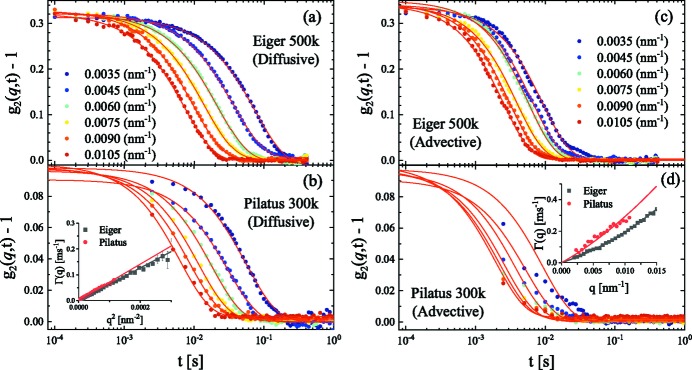
Comparison of *g*_2_(*q*, *t*) functions (for *q* values indicated in the legend) measured with the Eiger 500k and Pilatus 300k detectors when colloids are undergoing diffusive and advective motions. The left-hand panels correspond to diffusive dynamics below the phase separation temperature of the solvent and the solid red lines represent fit curves to equation (2) with *D*_0_ values of (*a*) 0.61 µm^2^ s^−1^ and (*b*) 0.70 µm^2^ s^−1^. The right-hand panels present the change to predominantly advective dynamics during the phase separation of the solvent and the corresponding Γ(*q*) incorporates both advective and diffusive terms (Dattani *et al.*, 2017 ▸). The resulting fit parameters are (*c*) *D*_0_ ≃ 0.63 µm^2^ s^−1^ and Δ*v* ≃ 11.3 µm s^−1^, and (*d*) *D*_0_ ≃ 0.63 µm^2^ s^−1^ (fixed) and Δ*v* ≃ 22.8 µm s^−1^. The insets in panels (*b*) and (*d*) present the *q* dependence of Γ(*q*) in the diffusive and advective cases.

Until now, we have presented purely diffusive dynamics of colloids in an aqueous medium. The main purpose of a high-frame-rate detector is to enable investigation of fast non-equilibrium dynamics. This is illustrated by using an example involving silica colloidal particles suspended in a phase-separating liquid mixture composed of 3-methylpyridine, water and heavy water (Dattani *et al.*, 2017 ▸). The system phase-separates into coexisting methylpyridine-rich and water-rich phases above a certain temperature (∼43°C) for a water to heavy water ratio of 1:4 by weight. Below this temperature, particles display diffusive behavior as in water, which is shown in Figs. 6(*a*) and 6(*b*) ▸. Upon phase separation of the solvent, silica particles migrate into the methylpyridine-rich phase. This migration process is manifested as a fast advective motion of colloids. Figs. 6(*c*) and 6(*d*) ▸ depict the accelerated dynamics of particles during the phase-separation process.

A comparison with the data obtained using a Pilatus 300k detector for a similar sample is shown. The Eiger 500k detector captured the full details of the *g*_2_(*q*, *t*) functions with good statistics, which is essential for deciphering the advective and diffusive contributions in the observed dynamics. The model fits in Figs. 6(*c*) and 6(*d*) ▸ incorporated both advective and diffusive terms in *g*_2_(*q*, *t*) (Dattani *et al.*, 2017 ▸). The advective term involves the average velocity fluctuations of the particles (Δ*v*) around their mean velocity and it has a linear *q* dependence. In addition, to improve the fits in Fig. 6(*c*) ▸, a compress exponent of 1.1 was included in equation (3), *i.e.* exp{−[2Γ(*q*)*t*]^1.1^}. The insets of Figs. 6(*b*) and 6(*d*) ▸ display a different *q* dependence of Γ(*q*) in the diffusive and advective cases, respectively. Γ(*q*) derived using the Eiger 500k detector clearly manifests the crossover from advective to diffusive dynamics as a function of *q*. Note that the Eiger and Pilatus data were obtained from two different samples at not exactly the same temperature which explains the differences in the fit parameters listed in the caption of Fig. 6 ▸ and the Γ(*q*) values plotted in insets of Figs. 6(*b*) and 6(*d*) ▸. This example demonstrates that multispeckle XPCS using the Eiger 500k detector allows fast advective motions to be probed in active colloidal systems. The high speckle contrast and time resolution enabled more reliable separation of advective and diffusive contributions in the observed dynamics.

## 5. Conclusion   

We have successfully implemented a fast Eiger 500k detector for USA-XPCS measurements at beamline ID02, ESRF. Synergy between the detector hardware and software developments were essential for achieving the highest performance of the detector. The high spatial and time resolution of the detector significantly improved the quality of the intensity autocorrelation functions and their intercept represented by β. The unique combination of ultra-small-angle and sub-millisecond acquisition broadens the size and time scales accessible by XPCS. As a result, dynamics much faster than diffusive motions can be investigated in aqueous suspensions over a broad size scale. This has opened the opportunity to probe sub-millisecond non-equilibrium dynamics in a variety of soft matter and biological systems either self-driven or imposed by an external hydrodynamic flow. Examples range from synthetic active colloids to biological cell motility. Higher energy of the measurements (12.5 keV) aids in reducing the radiation damage with soft matter and biological specimens.

The fast detector also enhances the throughput in sequential measurements such as those involved in X-ray speckle visibility spectroscopy (Verwohlt *et al.*, 2018 ▸). A larger number of pixels improves the signal-to-noise ratio when studying weakly scattering biological samples. XPCS can now be applied to radiation-sensitive biological systems by accumulating the statistics from scans at different spots on the sample. In combination with the upcoming extremely brilliant source, further enhancement in the performance of XPCS and related techniques is expected. This will allow closing the gap with neutron spin–echo spectroscopy in dynamic studies.

## References

[bb1] Als-Nielsen, J. & McMorrow, D. (2011). *Elements of Modern X-ray Physics.* New York: John Wiley and Sons.

[bb2] Berne, B. J. & Pecora, R. (2000). *Dynamic Light Scattering: with Applications to Chemistry, Biology, and Physics.* New York: Dover Publications.

[bb3] Brönnimann, C. & Trüb, P. (2016). *Synchrotron Light Sources and Free-Electron Lasers: Accelerator Physics, Instrumentation and Science Applications*, edited by E. J. Jaeschke, S. Khan, J. R. Schneider & J. Hastings, pp. 995–1027. Berlin: Springer.

[bb4] Caronna, C., Chushkin, Y., Madsen, A. & Cupane, A. (2008). *Phys. Rev. Lett.* **100**, 055702. 10.1103/PhysRevLett.100.055702 18352390

[bb5] Dattani, R., Semeraro, E. F. & Narayanan, T. (2017). *Soft Matter*, **13**, 2817–2822. 10.1039/c6sm02855a 28345703

[bb6] Dinapoli, R., Bergamaschi, A., Greiffenberg, D., Henrich, B., Horisberger, R., Johnson, I., Mozzanica, A., Radicci, V., Schmitt, B., Shi, X. & Tinti, G. (2013). *Nucl. Instrum. Methods Phys. Res. A*, **731**, 68–73.

[bb7] Dinapoli, R., Bergamaschi, A., Henrich, B., Horisberger, R., Johnson, I., Mozzanica, A., Schmid, E., Schmitt, B., Schreiber, A., Shi, X. & Theidel, G. (2011). *Nucl. Instrum. Methods Phys. Res. A*, **650**, 79–83.

[bb8] Goodman, J. (1985). *Statistical Optics.* New York: Wiley.

[bb9] Grübel, G., Madsen, A. & Robert, A. (2008). In *Soft-Matter Characterization*, edited by R. Borsali and R. Pecora, ch. 18. Berlin, Heidelberg: Springer-Verlag.

[bb10] Hoshino, T., Kikuchi, M., Murakami, D., Harada, Y., Mitamura, K., Ito, K., Tanaka, Y., Sasaki, S., Takata, M., Jinnai, H. & Takahara, A. (2012). *J. Synchrotron Rad.* **19**, 988–993. 10.1107/S0909049512038769 PMC3621499 23093759

[bb11] Johnson, I., Bergamaschi, A., Buitenhuis, J., Dinapoli, R., Greiffenberg, D., Henrich, B., Ikonen, T., Meier, G., Menzel, A., Mozzanica, A., Radicci, V., Satapathy, D. K., Schmitt, B. & Shi, X. (2012). *J. Synchrotron Rad.* **19**, 1001–1005. 10.1107/S0909049512035972 PMC3480275 23093761

[bb12] Leheny, R. L. (2012). *Curr. Opin. Colloid Interface Sci.* **17**, 3–12.

[bb13] Möller, J., Chushkin, Y., Prevost, S. & Narayanan, T. (2016). *J. Synchrotron Rad.* **23**, 929–936. 10.1107/S1600577516008092 PMC5315095 27359141

[bb14] Möller, J. & Narayanan, T. (2017). *Phys. Rev. Lett.* **118**, 198001. 10.1103/PhysRevLett.118.198001 28548515

[bb15] Narayanan, T. (2008). In *Soft-Matter Characterization*, edited by R. Borsali & R. Pecora, ch. 17. Berlin, Heidelberg: Springer-Verlag.

[bb16] Narayanan, T., Wacklin, H., Konovalov, O. & Lund, R. (2017). *Crystallogr. Rev.* **23**, 160–226.

[bb17] Petitdemange, S., Claustre, L., Henry, A., Homs, A., Homs Regojo, R., Langlois, F., Mant, G., Naudet, D., Noureddine, A., Papillon, E., Langlois, F., Mant, G. R. & Noureddine, A. (2018). *Proceedings of the 16th International Conference on Accelerator and Large Experimental Control Systems (ICALEPCS’17)*, Barcelona, Spain, 8–13 October 2017, pp. 886–890. TUPHA194.

[bb18] Sandy, A. R., Lurio, L. B., Mochrie, S. G. J., Malik, A., Stephenson, G. B., Pelletier, J. F. & Sutton, M. (1999). *J. Synchrotron Rad.* **6**, 1174–1184.

[bb19] Schavkan, A., Westermeier, F., Zozulya, A., Bondarenko, S., Grübel, G., Schroer, C. & Sprung, M. (2013). *J. Phys. Conf. Ser.* **425**, 202004.

[bb20] Sinha, S. K., Jiang, Z. & Lurio, L. B. (2014). *Adv. Mater.* **26**, 7764–7785. 10.1002/adma.201401094 25236339

[bb21] Sutton, M. (2008). *C. R. Phys.* **9**, 657–667.

[bb22] Tinti, G., Bergamaschi, A., Cartier, S., Dinapoli, R., Greiffenberg, D., Johnson, I., Jungmann-Smith, J., Mezza, D., Mozzanica, A., Schmitt, B. & Shi, X. (2015). *J. Instrum.* **10**, C03011.

[bb23] Verwohlt, J., Reiser, M., Randolph, L., Matic, A., Medina, L. A., Madsen, A., Sprung, M., Zozulya, A. & Gutt, C. (2018). *Phys. Rev. Lett.* **120**, 168001. 10.1103/PhysRevLett.120.168001 29756927

[bb24] Vodnala, P., Karunaratne, N., Lurio, L., Thurston, G. M., Vega, M., Gaillard, E., Narayanan, S., Sandy, A., Zhang, Q., Dufresne, E. M., Foffi, G., Grybos, P., Kmon, P., Maj, P. & Szczygiel, R. (2018). *Phys. Rev. E*, **97**, 020601. 10.1103/PhysRevE.97.020601 29548072

[bb25] Westermeier, F., Autenrieth, T., Gutt, C., Leupold, O., Duri, A., Menzel, A., Johnson, I., Broennimann, C. & Grübel, G. (2009). *J. Synchrotron Rad.* **16**, 687–689. 10.1107/S0909049509023280 19713644

[bb26] Zhang, Q., Dufresne, E. M., Grybos, P., Kmon, P., Maj, P., Narayanan, S., Deptuch, G. W., Szczygiel, R. & Sandy, A. (2016). *J. Synchrotron Rad.* **23**, 679–684. 10.1107/S1600577516005166 PMC5315006 27140146

